# Different distribution of adriamycin in normal and leukaemic rats.

**DOI:** 10.1038/bjc.1981.68

**Published:** 1981-04

**Authors:** P. Sonneveld, D. W. Van Bekkum

## Abstract

Adriamycin (ADR) accumulates in well-perfused organs in the rat. This effect is especially evident for long periods in marrow and spleen of healthy animals. In rats bearing the Brown Norway Acute Myeloid Leukaemia (BNML) the in vivo distribution is significantly different. Maximum ADR levels in those organs which are morphologically infiltrated by leukaemic cells are significantly lower than in normal rats, while the persistence of measurable ADR concentrations does not change. On the contrary, ADR concentrations in organs not infiltrated by leukaemic cells are the same or slightly higher than in normal rats. Possible causes for these differences are either the differential properties of normal and leukaemic cells in their uptake and excretion of ADR, or anatomical and vascular changes. It is evident that, significantly different from normal. This observation may help in the prevention of toxicity by drug monitoring in serum.


*Br. J. Cancer* (1981) **43**, 464

### DIFFERENT DISTRIBUTION OF ADRIAMYCIN IN NORMAL AND LEUKAEMIC RATS

P. SONNEVELD AND D. W. VAN BEKKUM

*From the Radiobiological Institute TNO, 2280 HV Rijswijk, The Netherlands*

Received 28 August 1980 Accepted 16 December 1980

**Summary.—Adriamycin (ADR) accumulates in well-perfused organs in the rat. This effect is especially evident for long periods in marrow and spleen of healthy animals. In rats bearing the Brown Norway Acute Myeloid Leukaemia (BNML) the *in vivo* distribution is significantly different. Maximum ADR levels in those organs which are morphologically infiltrated by leukaemic cells are significantly lower than in normal rats, while the persistence of measurable ADR concentrations does not change. On the contrary, ADR concentrations in organs not infiltrated by leukaemic cells are the same or slightly higher than in normal rats. Possible causes for these differences are either the differential properties of normal and leukaemic cells in their uptake and excretion of ADR, or anatomical and vascular changes. It is evident that, where leukaemia is involved, the relative distribution between serum and tissue is significantly different from normal. This observation may help in the prevention of toxicity by drug monitoring in serum.**

THE ANTHRAQUINONE GLYCOSIDE ADRIAMYCIN (ADR) is of widespread use in the treatment of solid tumours and acute leukaemia (Carter, 1975). Several studies on the distribution of the drug have revealed its rapid clearance from plasma, selective accumulation in various organs and slow elimination *via* urinary and biliary pathways in the rat, monkey and man (Chan *et al.*, 1978; Wilkinson *et al.*, 1979). Little information is available on the comparative distribution kinetics of ADR between normal tissues and malignant tumours *in vivo*. In mice and rats bearing solid tumours, it has been observed that ADR concentrations in metastases were higher than those in the parent tumours (Donelli *et al.*, 1977; Donelli & Garattini, 1977), while the drug penetrates less extensively into solid tumours than into most normal tissues (DiFronzo *et al.*, 1973). However, in acute leukaemia, a widely disseminated malignant disease, tumour cells are present in many organs in varying numbers. The differential drug uptake (Sonneveld & Van den Engh, 1981) and sensitivity (Buick *et al.*, 1979; Sonneveld *et al.*, 1981) between leukaemic and normal cells observed *in vitro* may thus very well influence the amount of drug taken up *in vivo* by an organ, especially if it is infiltrated by a large number of leukaemic cells. In view of the clinically observed variations in drug response among acute-leukaemia patients, the different drug uptake of normal and malignant cells, as well as the number of leukaemic cells, may be relevant factors in the *in vivo* distribution of ADR. The present study was initiated to investigate whether the distribution kinetics of ADR differ between normal and leukaemia-bearing rats using an experimental leukaemia model which has been proved to resemble closely acute myeloid leukaemia in man, and is highly sensitive to the antitumour activity of ADR.

### MATERIALS AND METHODS

*Leukaemia.*—The Brown Norway Myeloid

Requests for reprints to P. Sonneveld.

Leukaemia (BNML) was chosen as a model. This acute myelocytic leukaemia originated in 1971 in a female rat of the inbred Brown Norway rat strain in the Rijswijk colony (BN/Rij) following 3 i.v. injections of 2 mg of 9,10-dimethyl 1,2-benzanthracene 100 days earlier. The leukaemia has since been maintained by transplantation of leukaemic cells directly or by cryopreserved batches. The characteristics of BNML have been described extensively (Hagenbeek, 1977) and can be summarized as follows:

(1) the net growth rate is slow;

(2) it is cytochemically and cytologically identical to human acute myeloid (promyelocytic) leukaemia (AML);

(3) its response to antileukaemic chemotherapy is similar to that of AML;

(4) the mean survival time after i.v. inoculation of $10^7$ leukaemic spleen cells is 22 days;

(5) 2 single administrations of ADR (7·5 mg/kg, interval 7 days) increase the survival time by 14 days.

These characteristics have made BNML a particularly suitable model for the study of the proliferation kinetics and experimental treatment of acute leukaemia (Aglietta & Colly, 1979; Aglietta & Sonneveld, 1978). Therefore, the distribution kinetics of ADR were investigated in normal and BNML rats, the latter at a stage comparable with that of human AML patients at clinical admission (Day 15 after i.v. transplantation of $10^7$ leukaemic spleen cells). At this stage, marrow, spleen and liver are heavily infiltrated by leukaemic cells, and haemopoiesis is severely suppressed.

*Leukaemia transfer.*—The leukaemic spleen was always used as a source of leukaemic cells for transplantation. After resuspension of spleen cells obtained from a 21-day-old leukaemic rat in Hanks' balanced salt solution, the required number of cells was injected i.v. into a tail vein of the recipient in a final volume of 1 ml.

Distribution studies in leukaemic rats were generally performed at Day 15 after inoculation of $10^7$ leukaemic spleen cells.

*Animals.*—The studies were performed in the inbred BN rat strain from the Rijswijk colony. Female rats 12 weeks of age, bred under specific-pathogen-free (SPF) conditions, were used. Water and food were always supplied *ad libitum* during the experiments.

*Experimental drug.*—Adriamycin hydrochloride was kindly supplied by Farmitalia, Milan, Italy. The chemical purity was assessed by thin-layer chromatography, using Merck silica-gel glass discs. The solvent used was n-butanol, 60%; glacial acetic acid, 15% distilled water, 25%.

The chemical purity of the drug was 95%. No variations in purity were noted during dry storage of the drug at room temperature.

*Collection of biological specimens.*—In most experiments, ADR was administered into a tail vein at a dose of 7·5 mg/kg (44 mg/m$^2$ of body surface) (Freireich, 1966) after solution in sterile 0·9% NaCl at a final concentration of 2 mg/ml. To avoid a decrease in activity of the drug, it was always dissolved 2 h before each experiment.

To determine the concentrations in serum and organs, animals were killed at various intervals after i.v. administration of the drug. For each time interval, 4 normal, 4 leukaemic and 1 untreated animal were used. Animals were killed by ether anaesthesia and cervical dislocation. Blood was then obtained by aortic puncture. The blood (generally 4 ml) was centrifuged for 10 min at 400 *g*. After carefully pipetting off the upper phase, serum was kept at 4°C until chemical analysis was performed. Liver, spleen, kidneys, heart and lungs were removed from the body, weighed and homogenized immediately after addition of sterile 0·9% NaCl to obtain a final dilution of 10% v/v. Immediately after homogenization, the tissue samples were stored at 4°C. Marrow was obtained after the femurs had been freed of adherent muscle tissue; the proximal ends were cut with a surgical knife and the femoral shafts were emptied by repeated flushing with Hanks' solution using a needle on a 5ml syringe. The collected marrow was filtered and cells were counted microscopically using a Bürker type haemacytometer after staining with Türck's solution (0·01%).

*Extraction procedure.*—For deproteinizing the cell suspensions and extraction of ADR from serum and cell suspensions, the method described by Schwartz was used, because of its high tissue recovery (90%) of ADR (Schwartz, 1973). This method does not give rise to unwanted metabolism of the parent drug during the extraction.

Determination of the ADR concentration was performed spectrofluorometrically in a Hitachi-FL 204 fluorescence spectrophotometer. As a standard, samples from untreated animals were used and this background fluorescence was subtracted from experimental values.

In all experiments, the excitation wavelength was 460 nm and the samples were read at a fluorescence of 580 nm. Pure isoamyl alcohol served as the blank control.

*Renal function.* — Since Adriamycin is partly excreted in the urine, it is necessary to monitor renal function in experiments on the pharmacokinetics of the drug. The creatinine concentration of the blood, being a function of the renal excretory capacity, was ascertained in normal and in leukaemic rats at different stages of the disease. A standard commercial kit (Boehringer, Ingelheim) was used for spectrometric determinations.

*Serum volume.*—The serum volume was determined by injection of $^{14}$C-inulin (sp. act. 1–3 $\mu$Ci/mg inulin–carboxylic acid) at a final concentration of 10 $\mu$Ci in 1 ml. After 1 min, cardiac puncture was performed and the blood obtained was centrifuged, after clotting, at 700 *g* for 10 min. The serum was pipetted off and the radioactivity, expressed as c/min, was measured in scintillation vials (Packard Instr., Zurich, Switzerland) in a liquid scintillation counter (Nuclear Chicago, Mark II) using a toluene-based scintillation fluid (50 mg POPOP and 4 g PPO/l toluene). The serum volume was calculated by dividing the ct/min produced by 10 $\mu$Ci $^{14}$C-inulin by that produced by 1 ml of serum. The total blood volume was derived from this value by multiplying the serum volume by the reciprocal of the haematocrit.

### RESULTS

### *Distribution of ADR in normal and leukaemic rats*

Although the concentration–time curve in serum provides information on the extent of drug penetration into the tissues, selective accumulation can only be detected by measuring the drug concentration in various organs. Since a leukaemia model was chosen to study the distribution of ADR in the presence of tumour load, the following organs were selected for examination; liver, spleen and femoral marrow (as major sites of leukaemic infiltration), heart and lungs (as major sites for selective toxicity) and kidneys, because of their excretory function. Figs 1–3 and

FIG. 1.—Serum disappearance curve of Adriamycin following a rapid intravenous bolus injection of 7·5 mg/kg dissolved in 0·9% NaCl in a final concentration of 2 mg/ml in normal (——) and 15-day leukaemic (– – –) rats (± 2 s.e.).

FIG. 2.—Spleen concentration of Adriamycin in normal (——) and 15-day leukaemic (– – –) rats following 7·5 mg/kg i.v. (± 2 s.e.). The symbols represent the concentration per g of tissue.

Table II show the typical disappearance curves obtained after i.v. administration of 7·5 mg/kg in normal and in leukaemic rats at Day 15 after inoculation. At this stage, the mean weights of the organs investigated averaged the values indicated in Table I.

*Serum* (Fig. 1).—After i.v. administra-

TABLE I.—*Weights (g) of body and organs of normal and 15-day BNML rats*

| | BN | BNML |
|---|---|---|
| Body weight | 165·05 ± 3·10 | 165·85 ± 4·55 |
| Plasma | 5·70 ± 0·18 | 5·10 ± 0·18 |
| Spleen | 0·35 ± 0·11 | 1·28 ± 0·10 |
| Liver | 4·94 ± 0·25 | 8·50 ± 0·38 |
| Heart | 0·60 ± 0·04 | 0·58 ± 0·02 |
| Lungs | 1·35 ± 0·02 | 1·38 ± 0·03 |
| Kidneys | 0·96 ± 0·01 | 1·04 ± 0·02 |
| Skeleton | 10·40 ± 0·24 | |
| Skin | 35·00 ± 1·45 | |

The results represent the values (± s.d.) obtained in ~60 females of 12 weeks of age. (Values for the plasma volumes from Hagenbeek, 1977.)

tion a rapid initial decrease of the serum concentration of ADR can be seen in both normal and leukaemic rats followed by a long excretory phase. These concentration–time curves suggest extensive accumulation of the drug in the tissues. The values for leukaemic animals are consistently higher than those for normal rats. The observed differences are significant for a number of points.

*Spleen* (Fig. 2).—ADR levels in the spleen reach values 10× the serum concentration within 10 min of drug administration. Despite the initial decrease in the first 30 min, the spleen drug levels remain high for 24 h in normal rats. In leukaemic animals, in which the spleen weight has increased 5-fold and large numbers of leukaemic cells have infiltrated the organ, the drug levels are generally lower by a factor 1·5–2 (in the first 24 h) than those of normal rats.

*Marrow* (Fig. 3).—Considerable concentrations of ADR are found in the femoral bone marrow of both normal and leukaemic rats. In Fig. 3 the concentration–time curves are plotted of the first 8 h expressed as $\mu$g/$10^8$ nucleated cells. Assuming a specific density of 1·00 and a mean cell diameter of 8 $\mu$m, the weight of $10^8$ cells is 26 mg. This means that for comparative purpose the marrow concentrations of ADR may be expressed as $\mu$g/g of tissue by multiplying the concentrations per $10^8$ cells by a factor of 38·5.

The concentration–time curve of ADR in marrow is characterized by a gradual increase both in normal and leukaemic rats. However, in BNML rats the large presence of leukaemic cells apparently inhibits drug accumulation. In BN and BNML animals the observed peak concentrations in marrow are considerably higher than in the other organs investigated in this study.

*Other organs.*—Table II shows the disappearance of ADR in several organs which, as judged by microscopy, are hardly if at all infiltrated by leukaemic cells, such as lungs, heart, kidneys and liver. Except in liver, no significant differences can be seen between the concentration–time relationships in organs from normal and leukaemic organs. The disappearance of ADR from the liver

FIG. 3.—Concentration of Adriamycin in femoral marrow cells of normal (——) and 15-day leukaemic (– – –) rats following 7·5 mg/kg i.v. (± 2 s.e.).

FIG. 4.—Cumulative urinary excretion of Adriamycin following a single i.v. bolus injection of 7·5 mg/kg (± 2 s.e.) in normal and BNML rats.

TABLE II.—*Disappearance of ADR from organs of normal (BN) and leukaemic (BNML) rats following 7·5 mg/kg i.v. (μg/g tissue)*

| | Liver | | Heart | | Lungs | | Kidneys | |
|---|---|---|---|---|---|---|---|---|
| | BN | BNML | BN | BNML | BN | BNML | BN | BNML |
| 10 min | 32·0 | 29·2 | 13·9 | 14·0 | 16·7 | 20·0 | 38·7 | 34·6 |
| 20 min | 28·1 | 27·5 | 12·7 | 13·5 | 14·6 | 17·1 | 26·8 | 28·1 |
| 30 min | 19·2 | 18·8 | 14·5 | 13·9 | 19·1 | 16·9 | 29·3 | 24·8 |
| 1 h | 15·6 | 11·4 | 12·8 | 10·5 | 15·5 | 14·7 | 23·5 | 20·6 |
| 2 h | 12·0 | 9·7 | 10·6 | 9·5 | 13·1 | 12·7 | 21·9 | 19·4 |
| 4 h | 6·0 | 8·4 | 8·9 | 7·9 | 10·2 | 9·8 | 14·9 | 17·5 |
| 8 h | 6·1 | 7·8 | 6·1 | 8·1 | 8·5 | 10·8 | 13·2 | 17·2 |
| 12 h | 4·2 | 6·1 | 5·9 | 7·0 | 8·7 | 10·3 | 12·2 | 14·1 |
| 24 h | 3·9 | 5·2 | 6·2 | 5·1 | 8·6 | 8·9 | 12·7 | 12·3 |

seems to be slower in leukaemic than in normal rats.

*Renal elimination*

To determine the renal elimination of ADR, rats were caged in metabolic cages, in which urine is collected without the chance of pollution by faeces (Hagenbeek, 1977). Fig. 4 shows the cumulative renal elimination of ADR over 72 h in normal animals. The mean volume of urine in which this amount of ADR was excreted was 21 ml. The renal function as measured by the creatinine content of the serum changed significantly during progress of the disease, and was actually higher by a factor of 1·4 at Day 15 after inoculation of the leukaemia, than in healthy controls.

*Biliary excretion*

To determine the excretion of ADR in bile, 9 animals were anaesthetized with ketamine HCL and a teflon drain with a diameter of 1 mm was inserted into the biliary duct. The drain was led through the cutis and inserted into a 5ml tube. Saline (0·9%) was administered i.v. at a rate of 2 ml/h by an infusion pump during sampling in order to maintain the fluid balance. After the end of the anaesthesia, ADR was administered i.v. in a single dose and the bile was collected at regular intervals. In Fig. 5, the cumulative biliary elimination is plotted for 24 h. A rapid initial excretion can be observed for 6 h, after which the elimination diminishes. These observations are from healthy rats only, since leukaemic rats did not survive the surgery.

FIG. 5.—Cumulative biliary excretion of Adriamycin in normal rats following 7·5 mg/kg. The values represent the means of 9 animals (±2 s.e.).

*Metabolism*

Using the described TLC chromatographic procedure in the first 24 h after administration of the drug, no Adriamycin or Adriamycinone could be demonstrated in serum and urine of either normal or leukaemic animals. This finding accords with the results of Wilkinson *et al.* (1979), who found no metabolic breakdown of ADR in rats, using high-performance liquid chromatography.

### DISCUSSION

It is not possible to ascertain from the serum curve which organs excessively accumulate ADR. Careful analysis of the distribution of the agent in several organs is therefore needed in order to discover where ADR accumulates and how long it remains in each organ.

Analysis of the serum disappearance curve after 7·5 mg/kg i.v. reveals that, after 10 min, the concentration is 1·41 μg/ml. Bearing in mind that a total of 1240 μg was injected into a rat of 165 g, and that the serum volume amounts to 5·7 ml, the conclusion is that ADR is redistributed very rapidly to the tissues. Calculated per g of tissue, a great part of the administered dose accumulates in the marrow cells of both BNML and normal rats. In view of the severe toxicity of ADR to the normal haemopoietic system, which is located in the marrow, this toxicity is not only due to the haemopoietic system being composed of rapidly dividing cells, but the high concentration over a prolonged period may also be responsible. In leukaemic animals, the marrow concentration is considerably lower. Leukaemic cells, which contribute greatly to the population of the femoral marrow, probably take up ADR less than do normal haemopoietic cells.

A similar phenomenon can be observed in the spleen; during the 24h observation period, the concentration per g of spleen tissue is relatively constant in both normal and 15-day leukaemic rats. However, the concentration in leukaemic spleen is significantly lower, but shows the same disappearance pattern as the normal intracellular spleen concentration of normal spleen cells. In view of the increase in the spleen weight from 0·35 g to 1·28 g at Day 15, reduced availability of ADR to the individual cells in the spleen as a result of pathological deviations of the vascular structure should be considered as a possible causative factor. This reduction resembles that seen in marrow infiltrated with large numbers of leukaemic cells. Although the total ADR in a leukaemic spleen is greater than in the normal spleen, the concentration per cell is lower. At the same time, an increase in serum concentration can be seen during the first 8 h. A redistribution of ADR presumably occurs when a certain number of tumour cells is present in the body. In that case, it is impossible to estimate the concentration of ADR in the spleen from the serum curve, since there is no information on the exact relationship between these two organs.

The liver, which increases in weight by a factor of 1·72, shows only moderate deviations from the original concentration–time curve. Other organs such as the lungs, the heart and the kidneys show the same disappearance curve in normal and leukaemic animals. On histological examination, no leukaemic infiltration into these organs can be demonstrated and no increase in weight is observed (Hagenbeek, 1977). The quantitative differences between the serum curves of BN and BNML rats can be attributed to the lower drug levels in leukaemia-infiltrated organs: spleen and marrow.

Previously, Donelli (1976) had found reduced levels of ADR in tumour-infiltrated mouse lungs, when compared with normal lungs. In these animals, the excretion of ADR occurs according to the pathways which have been described (Bachur, 1975; Benjamin *et al.*, 1973, 1974). Since the contribution of an enterohepatic recirculation is not defined, it is uncertain how far the results can be compared. An 8% biliary elimination in 24 h, compared with a 50% excretion in the faeces in 7 days, does not provide strong evidence for the relevance of such an enterohepatic circulation. The urinary route is the major pathway of excretion in the normal rat (26% in 72 h). However, the decrease of normal renal elimination function, as measured by the creatinine content of peripheral blood, causes a decreased excretion of ADR in leukaemic rats. This decrease in excretory function leads to prolonged elevated levels of ADR in all normal tissues of leukaemic rats. A great proportion of the effective tumour-reducing function and the toxicity to normal tissues is probably a direct result of this phenomenon, especially since ADR elimination from many organs is slow.

Our results indicate significant differences in the general distribution of ADR between normal and leukaemic animals. These differences cannot be quantitated merely from the serum curve. In both normal and leukaemic animals, various organs show selective accumulation of the drug. It can be concluded, therefore, that an estimation of the drug level at the tumour site cannot be made without information on the tumour load and the normal drug distribution.

The author is indebted to Mrs W. Asscheman-Heus and Mr J. Vollebregt for expert technical assistance. The gift of Adriamycin from Farmitalia, Milan, Italy, is gratefully acknowledged.

This study was supported by the Koningin Wilhelmina Fonds of the Dutch National Cancer League.

### REFERENCES

AGLIETTA, M. & COLLY, L. P. (1979) The relevance of recruitment-synchronization in the scheduling of ara-C in a slow growing acute myeloid leukemia of the rat. *Cancer Res.*, **39**, 2727.

AGLIETTA, M. & SONNEVELD, P. (1978) The relevance of cell kinetics for optimal scheduling of 1-$\beta$-D-Arabinofuranosyl cytosine and methotrexate in a slow growing acute myeloid leukemia (BNML). *Cancer Chemother. Pharmacol.*, **1**, 219.

BACHUR, N. R. (1975) Adriamycin (NSC-123127) pharmacology. *Cancer Chemother. Rep.*, **3**, 153.

BENJAMIN, R. S. (1975) Clinical pharmacology of Adriamycin (NSC-123127). *Cancer Chemother. Rep.*, **3**, 183.

BENJAMIN, R. S., RIGGS, C. E. & BACHUR, N. R. (1973) Pharmacokinetics and metabolism of Adriamycin in man. *Clin. Pharmacol. Ther.*, **14**, 592.

BENJAMIN, R. S., RIGGS, C. E. & SERPICK, A. A. (1974) Biliary excretion of Adriamycin (A) in man. *Clin. Res.*, **22**, 483.

BUICK, R. N., MESSNER, H. A., TILL, J. E. & MCCULLOUCH, E. A. (1979) Cytotoxicity of Adriamycin and Daunorubicin for normal and leukemia progenitor cells of man. *J. Natl Cancer Inst.*, **62**, 249.

CARTER, S. K. (1975) Adriamycin, a review. *J. Natl Cancer Inst.*, **55**, 1265.

CHAN, K. K., COHEN, J. L., GROSS, J. F. & 4 others (1978) Prediction of Adriamycin disposition in cancer patients using a physiologic, pharmacokinetic model. *Cancer Treat. Rep.*, **62**, 1161.

DIFRONZO, G., LENAZ, L. & BONADONNA, G. (1973) Distribution and excretion of Adriamycin in man. *Biomed. Express*, **19**, 169.

DONELLI, M. G., COLOMBO, T., BROGGINI, M. & GARATTINI, S. (1977) Differential distribution of antitumor agents in primary and secondary tumors. *Cancer Treat. Rep.*, **61**, 1319.

DONELLI, M. G. & GARATTINI, S. (1977) Differential accumulation of anticancer agents in metastases compared with primary tumors in experimental models. In *Recent Advances in Cancer Treatment.* Eds Tagnon & Staquet. New York: Raven Press. p. 177.

DONELLI, M. G., MARTINI, A., COLOMBO, T., BOSSI, A. & GARATTINI, S. (1976) Heart levels of adriamycin in normal and tumor-bearing mice. *Eur. J. Cancer*, **12**, 913.

FREIREICH, E. J. (1966) Quantitative comparison of toxicity of anticancer agents in mouse, rat, dog, monkey and man. *Cancer Chemother. Rep.*, **50**, 219.

HAGENBEEK, A. (1977) Extracorporeal irradiation of the blood in a rat leukemia model. *Thesis Erasmus University, Rotterdam.*

SCHWARTZ, H. S. (1973) A fluorometric assay for Daunomycin and Adriamycin in animal tissues. *Biochem. Med.*, **7**, 396.

SONNEVELD, P. & VAN DEN ENGH, G. J. (1981) *Leukemia Res.* (In press.)

SONNEVELD, P., MULDER, J. A. & VAN BEKKUM, D. W. (1981) *Cancer Chemother. Pharmacol.* (In press.)

WILKINSON, P. M., ISRAEL, M., PEGG, W. J. & FREI, E. III (1979) Comparative metabolism and excretion of Adriamycin in man, monkey and rat. *Cancer Chemother. Pharmacol.*, **2**, 121.


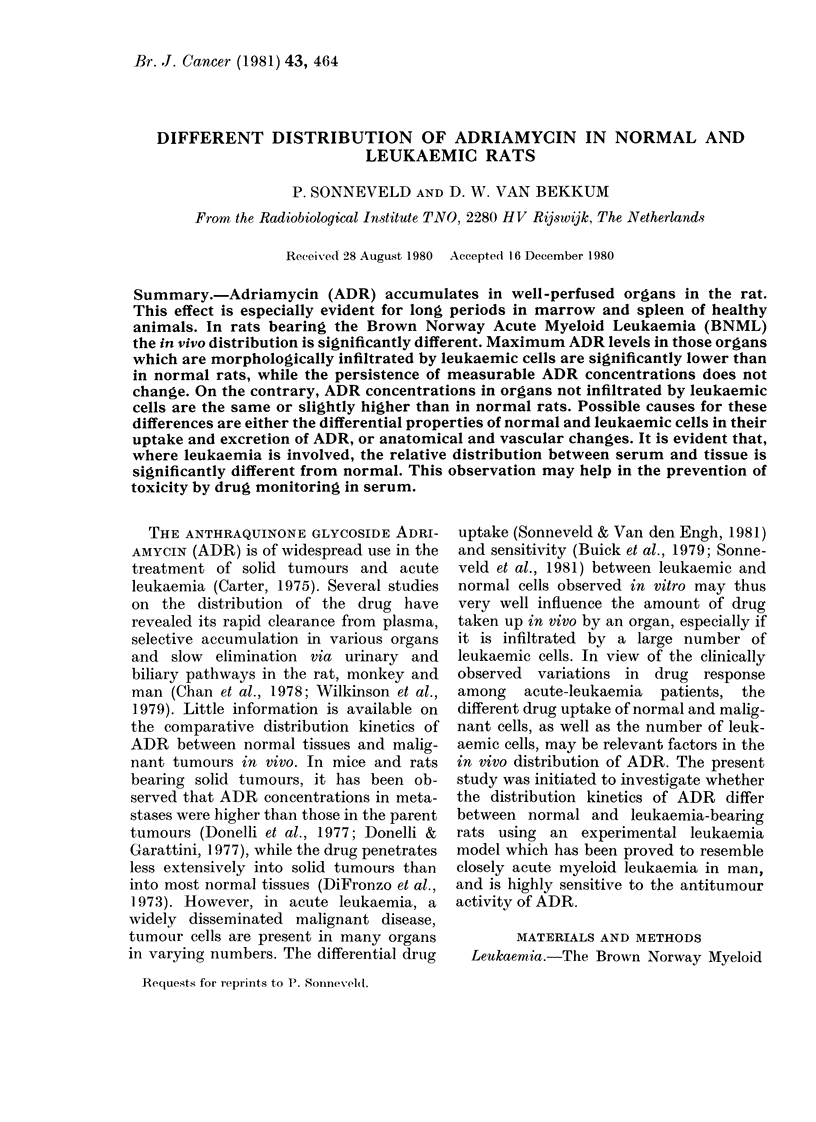

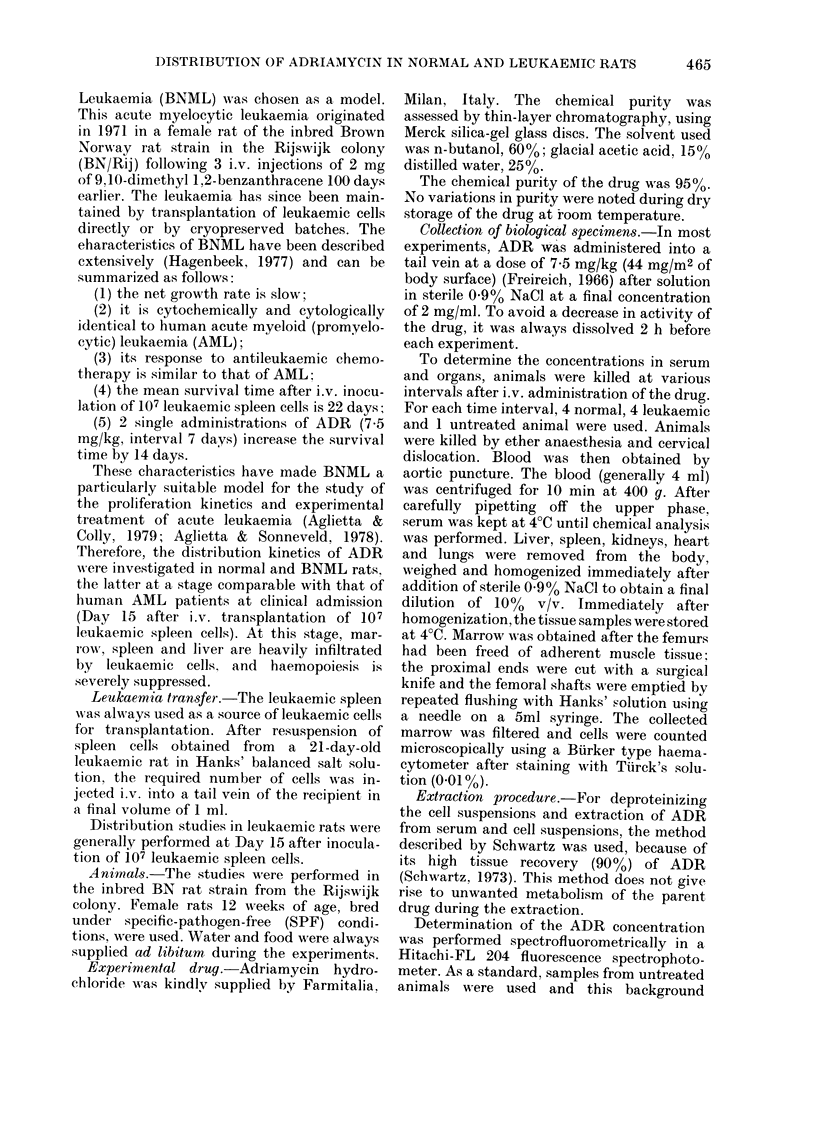

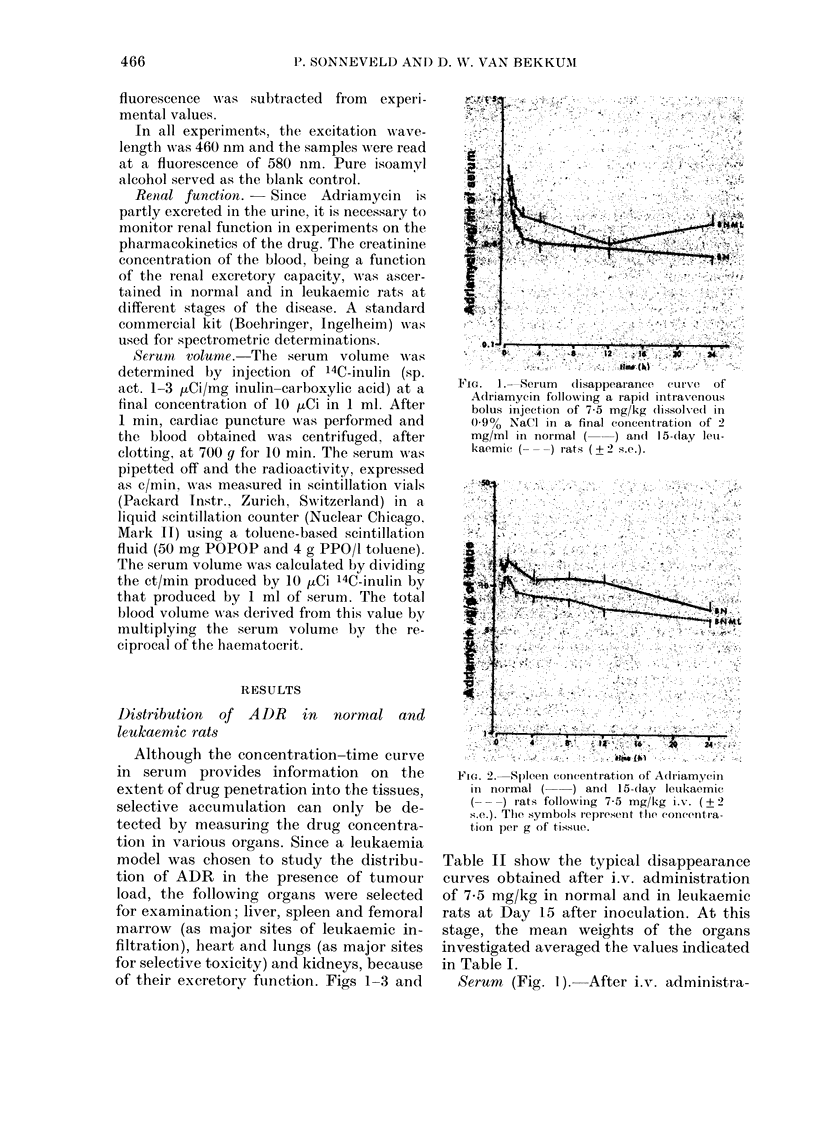

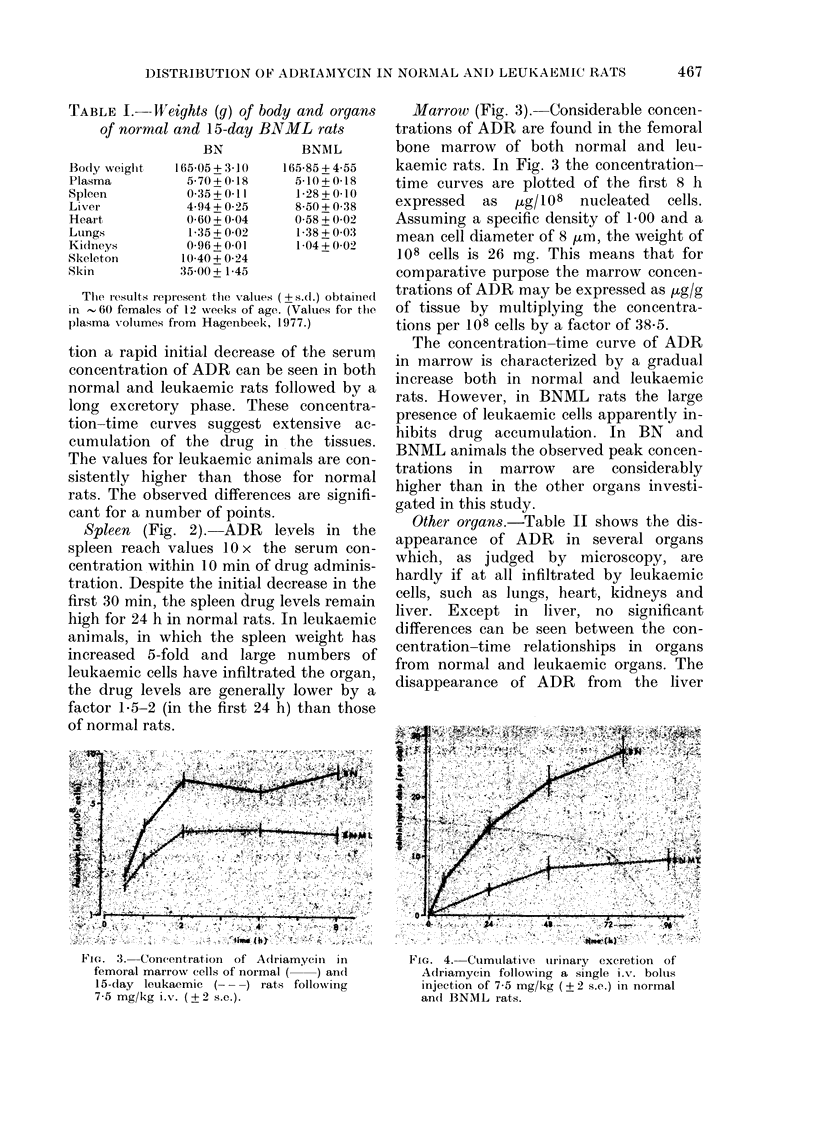

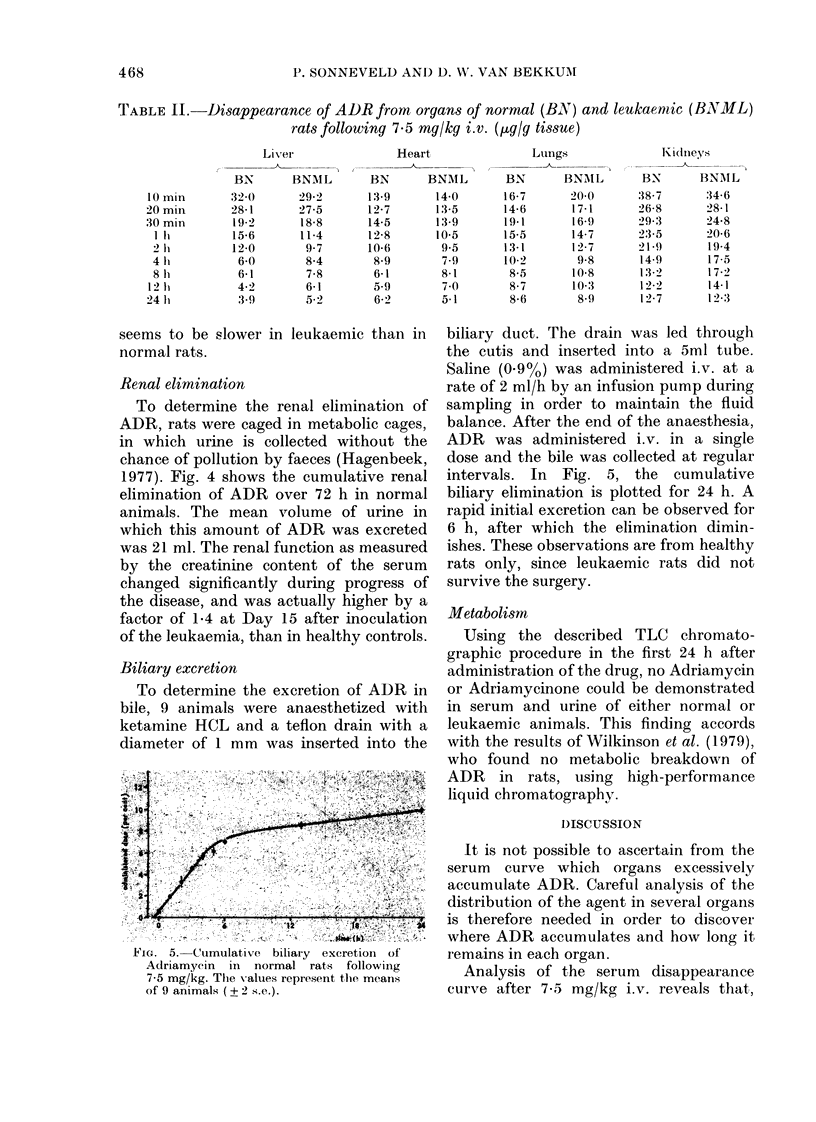

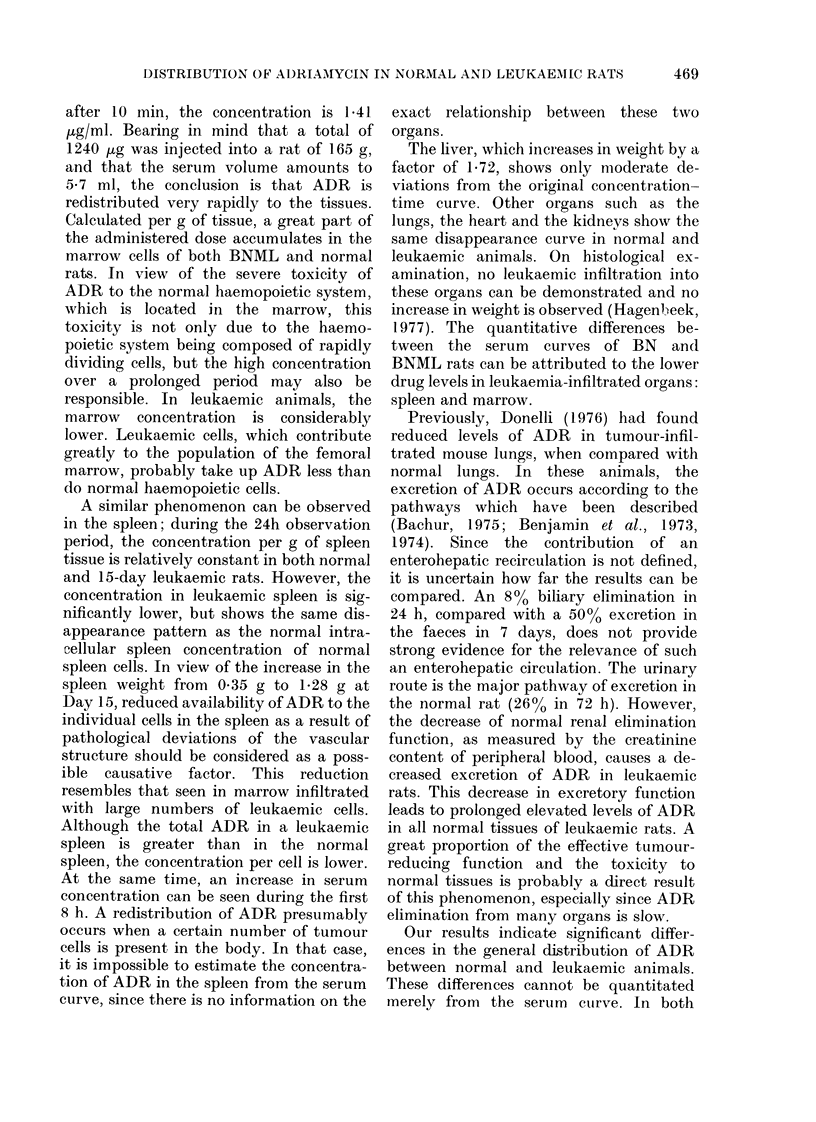

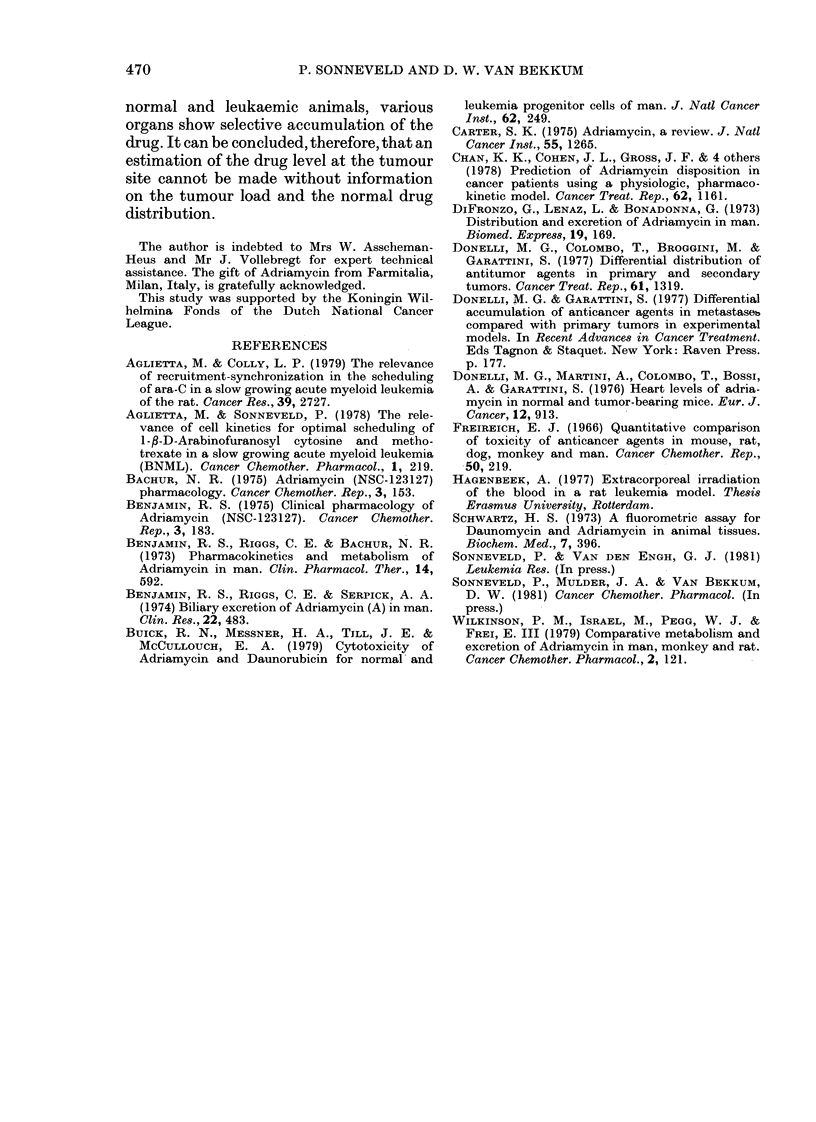


## References

[OCR_00794] Aglietta M., Colly L. (1979). Relevance of recruitment-synchronization in the scheduling of 1-beta-D-arabinofuranosylcytosine in a slow-growing acute myeloid leukemia of the rat.. Cancer Res.

[OCR_00800] Aglietta M., Sonneveld P. (1978). The relevance of cell kinetics for optimal scheduling of 1-beta-D-arabinofuranosyl cytosine and methotrexate in a slow growing acute myeloid leukemia (BNML).. Cancer Chemother Pharmacol.

[OCR_00815] Benjamin R. S., Riggs C. E., Bachur N. R. (1973). Pharmacokinetics and metabolism of adriamycin in man.. Clin Pharmacol Ther.

[OCR_00826] Buick R. N., Messner H. A., Till J. E., McCulloch E. A. (1979). Cytotoxicity of adriamycin and daunorubicin for normal and leukemia progenitor cells of man.. J Natl Cancer Inst.

[OCR_00834] Carter S. K. (1975). Adriamycin-a review.. J Natl Cancer Inst.

[OCR_00838] Chan K. K., Cohen J. L., Gross J. F., Himmelstein K. J., Bateman J. R., Tsu-Lee Y., Marlis A. S. (1978). Prediction of adriamycin disposition in cancer patients using a physiologic, pharmacokinetic model.. Cancer Treat Rep.

[OCR_00849] Donelli M. G., Colombo T., Broggini M., Garattini S. (1977). Differential distribution of antitumor agents in primary and secondary tumors.. Cancer Treat Rep.

[OCR_00865] Donelli M. G., Martini A., Colombo T., Bossi A., Garattini S. (1976). Heart levels of adriamycin in normal and tumor-bearing mice.. Eur J Cancer.

[OCR_00872] Freireich E. J., Gehan E. A., Rall D. P., Schmidt L. H., Skipper H. E. (1966). Quantitative comparison of toxicity of anticancer agents in mouse, rat, hamster, dog, monkey, and man.. Cancer Chemother Rep.

[OCR_00884] Schwartz H. S. (1973). A fluorometric assay for daunomycin and adriamycin in animal tissues.. Biochem Med.

[OCR_00900] Wilkinson P. M., Israel M., Pegg W. J., Frei E. (1979). Comparative metabolism and excretion of adriamycin in man, monkey, and rat.. Cancer Chemother Pharmacol.

